# Interaction analysis of *Mycobacterium tuberculosis* between the host environment and highly mutated genes from population genetic structure comparison

**DOI:** 10.1097/MD.0000000000027125

**Published:** 2021-09-03

**Authors:** Zhezhe Cui, Jun Liu, Yue Chang, Dingwen Lin, Dan Luo, Jing Ou, Liwen Huang

**Affiliations:** a Department of Tuberculosis Control, Guangxi Zhuang Autonomous Region Center for Disease Control and Prevention, Nanning, Guangxi, China; b Department of Neurosurgery, Liuzhou People's Hospital, Liuzhou, Guangxi, China; c School of Medicine and Health Management, Guizhou Medical University, Guiyang, Guizhou, China; d Department of Biostatistics, Public Health and Management, Guangxi University of Chinese Medicine, Nanning, China.

**Keywords:** gene, host environment, interaction, mutation, tuberculosis

## Abstract

We aimed to investigate the genetic and demographic differences and interactions between areas where observed genomic variations in Mycobacterium tuberculosis (*M. tb)* were distributed uniformly in cold and hot spots.

The cold and hot spot areas were identified using the reported incidence of TB over the previous 5 years. Whole genome sequencing was performed on 291 *M. tb* isolates between January and June 2018. Analysis of molecular variance and a multifactor dimensionality reduction (MDR) model was applied to test gene-gene-environment interactions. Adjusted odds ratios (OR) and 95% confidence intervals (CI) were computed to test the extent to which genetic mutation affects the TB epidemic using a multivariate logistic regression model.

The percentage of the Beijing family strain in hot spots was significantly higher than that in cold spots (64.63% vs 50.69%, *P* = .022), among the elderly, people with a low BMI, and those having a history of contact with a TB patient (all *P* < .05). Individuals from cold spot areas had a higher frequency of out-of-town traveling (*P* < .05). The mutation of Rv1186c, Rv3900c, Rv1508c, Rv0210, and an Intergenic Region (SNP site: 3847237) showed a significant difference between cold and hot spots. (*P* < .001). The MDR model displayed a clear negative interaction effect of age groups with BMI (interaction entropy: −3.55%) and mutation of Rv0210 (interaction entropy: −2.39%). Through the mutations of Rv0210 and BMI had a low independent effect (interaction entropy: −1.46%).

Our data suggests a statistically significant role of age, BMI and the polymorphisms of Rv0210 genes in the transmission and development of *M. tb*. The results provide clues for the study of susceptibility genes of *M. tb* in different populations. The characteristic strains showed a local epidemic. Strengthening genotype monitoring of strains in various regions can be used as an early warning signal of epidemic spillover.

## 1 Introduction

Tuberculosis (TB) is still considered to be the major respiratory infectious disease affecting human health and seriously hindering economic development in developing countries. The global TB report shows that the estimated number of cases in 2019 was 10 million^[1]^ and China accounted for 9% of all new cases. Guangxi is a province with a relatively high incidence of TB compared to other provinces, but the reported incidence is spatially heterogeneous. Differences in reported morbidity rates across the province exceed 80/100,000 population. Regions, where observed genomic variations in Mycobacterium tuberculosis (*M. tb*) are distributed uniformly (cold spots) or localized to some region (hot spots), have been previously reported in Guangxi.^[2]^ Despite the reported correlation between TB reported incidence and environmental factors such as sunshine duration, GDP per capita and health insurance coverage, the correlation was relatively weak. The contribution of pathogen transmissibility and pathogenicity to the outbreak and its interaction with the environment are not well understood.

*M. tb* is the pathogen of TB and belongs to the *Mycobacterium tuberculosis* complex (MTBC). *M.tb* has high genetic homogeneity with other members of MTBC, and its sequence similarity at the gene level is more than 99.95%.^[3]^ However, the MTBC of different types has large differences in phenotype, pathogenicity and host tropism. Based on gene alignment studies, at least 20 regions among the genomes of the MTBC may have insertion/deletion events. Bacille Calmette-Guérin (BCG), the only TB vaccine available currently, is derived from mycobacterium *bovis* (M. *bovis*).^[4]^ A comparative analysis indicated that 14 regions of difference (RD 1–14), having indels or frameshift deletions, were absent in the BCG vaccine, compared to *M.tb* H37Rv (INSDC accession AL123456.3).^[5–7]^

Host-pathogen interaction and co-evolution are the results of species adaptation.^[8]^ Host immune pressure and pathogen immune evasion are key points in this process.^[9]^ Consistent with this concept, RD1, which is deleted in *M. bovis* BCG, encoded a type-VII secretion system and host immune response.^[10,11,7]^ Deletion of the RD2 gene also resulted in a decrease in the virulence of *M. tb*. However, this attenuation of virulence can in part be complemented by the introduction of the genes Rv1979c to 1982, but not other genes of RD2.^[12]^ Similarly, another study suggests that RD4 plays an important role in *mycobacterium* virulence, and RD4 knock-in BCG strains can provide a better protection.^[13]^

The biological and social behavior characteristics of the host may have a certain impact on the immune system of the body and affect the pathogenicity of *M. tb*. However, the effect of host-pathogen synergy on TB transmission is not clear. To further investigate the genetic and demographic differences between cold and hot spots of the TB epidemic and their interactions, and to propose hypotheses of TB transmission and virulence, we used a SNP-based method of whole-genome structural differences comparative analysis for highly mutated gene loci detection. We also examined whether these genes interacted with host environmental factors.

## 2 Methods

### 2.1 Subjects

Moran's I local spatial autocorrelation statistics, and space-time scan statistics were recruited to detect temporal and spatial clusters of tuberculosis reported incidence in Guangxi from 2010 to 2016.^[2]^ The spatiotemporal analysis identified 3 counties located in central Guangxi with a significant high TB-reported incidence cluster (hot spots), and 3 counties located in eastern Guangxi with a significant low TB-reported incidence cluster (cold spots). TB patients were confirmed with chest-X ray, sputum smear, culture, and drug sensitivity test from the TB designated hospitals of these 6 counties and were enrolled in this study with informed consent from January to June 2018. To be eligible for this cross-sectional study, participants must have been a resident in the study sites for at least 2 years and their isolates available for analysis. Children under the age of 5 and people with mental illness were excluded. According to the requirements of the national TB control program, all TB patients should be referred to designated hospitals at the county level for diagnosis and treatment. Through investigation, it was found that the rate of TB centralized treatment was more than 90%,^[14]^ so the data in this study were representative to a certain extent.

The sample size of this study was calculated based on the formula for comparing 2 independent proportions.^[15]^ According to a previous study, it was estimated that the mutation rate of the major SNP locus in the hot spots’ strains was 60% in hot spots and 40% in cold spots. On account of type I error of 0.05 and a power of 90%, at least 260 TB patients (130 cases in each area) and their sputum cultures were required.

### 2.2 Deoxyribonucleic acid extraction and whole genome sequencing

The sputum samples collected from the participants were cultured in the hospital of each study site. All isolates from the culture were transported to the Guangxi center for disease control and prevention for *M. tb* genomic deoxyribonucleic acid (DNA) extraction. The process followed the standard laboratory protocol (HiPure Bacterial DNA Kit, Magen Biotech Co. Ltd) and was stored at −80°C until sequencing. After quality assessment by Qubit 2.0 Fluorometer (Invitrogen, Carlsbad, CA, USA), next-generation sequencing library (350-base-pair) (bp) preparations were built for purified DNA based on the manufacturer's standard (Illumina TruSeq DNA Nano Library Prep Kit). Libraries with different indices were multiplexed and loaded on an Illumina HiSeq instrument following the instructions (Illumina, San Diego, CA, USA). The Burrows-Wheeler transform algorithm and genome analysis toolkit packages (GATK v 4.1.1.0, Broad Institute, USA) were employed for multiple sequence alignment test strains of SNPs/InDels. We performed all of the whole genome sequencings at least twice to validate the reproducibility.

### 2.3 Variables

Host variables obtained from participants included sex, age, ethnicity, monthly income, body mass index (BMI), history of household contact with another TB patient, history of smoking and drinking, history of BCG vaccination, travel history (For more than 3 months within 2 years in an area other than the place of permanent residence) and status of multi-drug resistance (MDR). Structured questionnaires were used to collect information about host data. Pathogen variables included mutation information of all SNP positions among included isolates. Cases with missing gene sequence data will be eliminated.

### 2.4 Statistical analyzes

Comparison of demographic characteristics and area of residence (cold vs hot spots) was done using Pearson's Chi-Squared test for categorical variables and the rank-sum test for continuous variables. Molecular typing and statistical inference were conducted based on the genotype assignment of the isolates based on the SNP classification.^[16]^ Analysis of Molecular Variance was used to test the genomic differentiation (F-statistic index) between the groups.^[17]^ MDR models were applied to test gene-gene-environment interactions.^[18]^ The best model was selected based on the prediction error using 10-fold cross-validations. Hierarchical interaction graphs and interaction dendrograms of MDR were employed to visualize the interactions of *M.tb* gene-host environment in the best model.^[19]^

## 3 Results

### 3.1 General situation

Thirteen objects were excluded, of whom 8 cases were *non-tuberculosis mycobacteria* infection and 5 cases had the low quality of DNA. Totally, 291 TB patients and their isolates were included in the study. There were 147 isolates in the 3 hot spot areas and 144 in the 3 cold spot areas. Through whole genome sequencing and molecular typing, the dominant strains in both areas belonged to the Beijing family (SPOLIGO typing, SITVIT2 Database). However, the proportion of Beijing family strains in hot spots was significantly higher than that in cold spots (64.6% vs 50.7%, *P* = .022). Other genotypes included T1, T2, T3, H, H3, and LAM which belong to the Euro-American lineage.

### 3.2 Comparison of demographic characteristics

As shown in Table 1, the elderly, ethnic minorities (91.5% were Zhuang), those with low income, low BMI and a history of contact with a former TB patient were the predominant characteristics of TB patients in hot spot areas (*P* < .05). Individuals from cold spot areas had a higher frequency of out-of-town traveling (*P* < .05). However, due to the obvious imbalance of the ethnicity and economic levels between cold and hot spots, the variables of ethnicity and income are not comparable.^[20]^

**Table 1:** Comparison of demographic characteristics of in TB cold and hot spots.

Variables	Cold spot (n%)	Hot spot (n%)	*P* value
Age_ group			
<30	35 (24.3)	10 (6.8)	<.001
30–49	40 (27.8)	47 (32.0)	
≥50	69 (47.9)	90 (61.2)	
Gender			
Male	114 (79.2)	113 (76.9)	.74
Female	30 (20.8)	34 (23.1)	
Ethnicity			
Han	139 (96.5)	5 (3.4)	<.001
Others^∗^	5 (3.5)	142 (96.6)	
Income (Yuan)			
<3000	100 (72.5)	133 (91.1)	<.001
3000–4999	21 (15.2)	12 (8.2)	
≥5000	17 (12.3)	1 (0.7)	
Migration			
No	110 (76.4)	129 (87.8)	.017
Yes	34 (23.6)	18 (12.2)	
TB patient contact history			
No	130 (91.5)	118 (80.8)	.014
Yes	12 (8.5)	28 (19.2)	
BMI			
<18	11 (7.6)	39 (26.5)	<.001
18–24.99	127 (88.2)	100 (68)	
≥25	6 (4.2)	8 (5.4)	
BCG vaccination			
No	18 (12.5)	23 (15.6)	.624
Yes	112 (77.8)	113 (76.9)	
Unknown	14 (9.7)	11 (7.5)	
History of DM			
No	122 (84.7)	111 (75.5)	.134
Yes	21 (14.6)	33 (22.4)	
Unknown	1 (0.7)	3 (2)	
Drinking status			
Never	98 (68.1)	87 (59.2)	.289
Former	28 (19.4)	36 (24.5)	
Current	18 (12.5)	24 (16.3)	
Smoking status			
Never	73 (50.7)	70 (47.6)	.696
Former	48 (33.3)	48 (32.7)	
Current	23 (16.0)	29 (19.7)	
Status of MDR			
Yes	2	6	.296
No	142	141	

∗ More than 95% were from the Zhuang ethnic minority group. BCG = Bacillus Calmette–Guérin. BMI = body mass index, DM = diabetes mellitus, MDR = multi-drug resistance, TB = tuberculosis.

### 3.3 Population genetic structure differences

**Figure 1:**
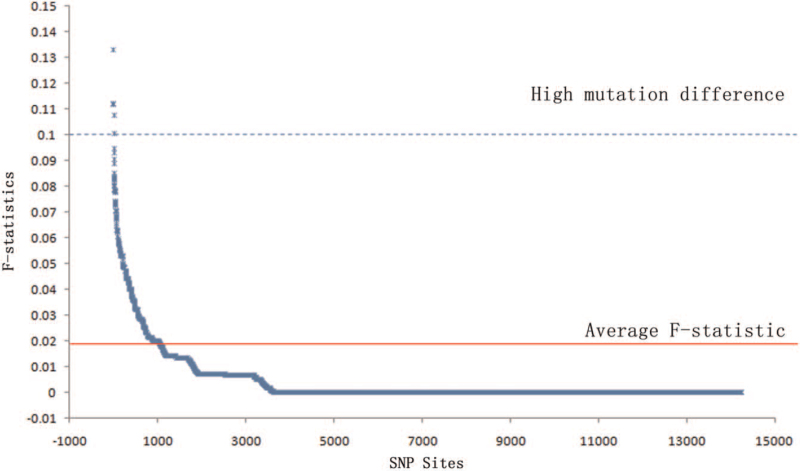
Fixed index (F-statistics) distribution of *M. tb* mutations between cold and hot spot areas.

Figure 1 shows a fixed index frequency distribution of mutations in cold and hot spots for various tuberculosis strains. The distribution represents the information of 14,250 mutated gene loci in 2 regions extracted from the SNPs file for molecular variance analysis. After filtering and Weir-Cockerham weighting, the average fixed index of the 2 groups was 0.019462. The fixed index of 5 SNP sites (1,328,687, 4,386,228, 3,847,237, 1,699,849, 251,575) was greater than 0.1, indicating that the mutation difference in these sites between the 2 populations was significant. Table 2. shows the gene locus and their gene products of these SNP sites. Just 1 SNP site (3847237) locates in the Intergenic Region.

**Table 2:** The information of 5 SNP sites with high fixed index.

Reference sequence	SNP position	F	Gene locus	Gene product
AL123456.3	1328687	0.133141	Rv1186c	Conserved protein
AL123456.3	4386228	0.11225	Rv3900c	Conserved hypothetical alanine rich protein
AL123456.3	3847237	0.111604	IGR^∗^	
AL123456.3	1699849	0.107462	Rv1508c	Probable membrane protein
AL123456.3	251575	0.100608	Rv0210	Hypothetical protein

∗ IGR = intergenic region.

As shown in Table 3, all the mutations of SNP site showed a significant difference between TB cold and hot spots. The proportion of 1,328,687 (Rv1186c) mutation was significant high in cold spots (OR = 0.32, 95%: 0.2–0.52), and the proportion of 4,386,228 (Rv3900c) (OR = 2.79, 95%: 1.74–4.5), 3847237 (IGR) (OR = 2.84, 95%: 1.75–4.62), 1699849 (Rv1508c) (OR = 2.73, 95%: 1.7–4.) and 251575 (Rv0210) (OR = 2.65, 95%: 1.65–4.27) was significant high in hot spots.

**Table 3:** Comparison of high-difference SNP sites.

SNP locus	Cold spot (n%)	Hot spot (n%)	Odds ratio (OR)	*P* value
1328687 (Rv1186c)				
None (Ref.)	46 (31.9)	87 (59.2)	0.32 (0.2,0.52)	<.001
Mutation	98 (68.1)	60 (40.8)		
4386228 (Rv3900c)				
None (Ref.)	85 (59)	50 (34)	2.79 (1.74,4.5)	<.001
Mutation	59 (41)	97 (66)		
3847237 (IGR)				
None (Ref.)	103 (71.5)	69 (46.9)	2.84 (1.75,4.62)	<.001
Mutation	41 (28.5)	78 (53.1)		
1699849 (Rv1508c)				
None (Ref.)	95 (66)	61 (41.5)	2.73 (1.7,4.4)	<.001
Mutation	49 (34)	86 (58.5)		
251575 (Rv0210)				
None (Ref.)	81 (56.2)	48 (32.7)	2.65 (1.65,4.27)	<.001
Mutation	63 (43.8)	99 (67.3)		

### 3.4 Multifactor dimensionality reduction analysis of gene-host environment interaction

**Figure 2:**
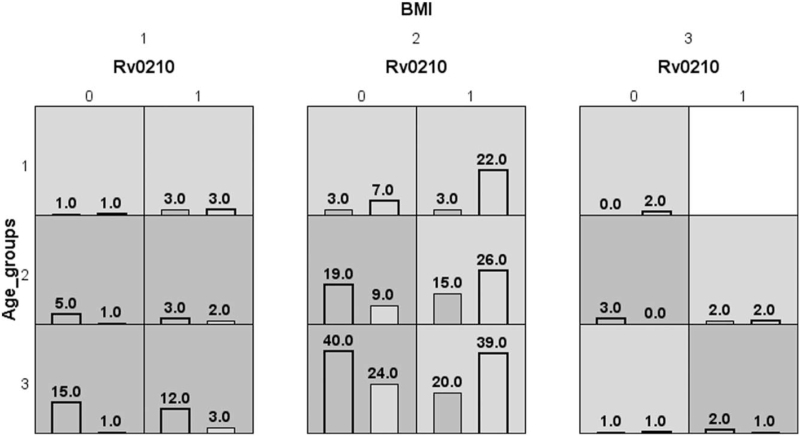
Distribution of high-risk and low-risk genotypes in the best model. Dark gray and light gray boxes represent high and low-risk factor combinations, respectively. Bars on the left within each box represent hot spots while those on the right represent cold spots. The numbers 0 and 1 appearing at the top and left of each box represent the classification of variables, respectively. The heights of the bars are proportional to the sample size in each group.

To test the gene-host environment interaction, the 5 SNP locus mentioned above and factors with significant differences between the 2 spots were included in the model. But ethnicity and income are excluded because of their incomparability. Table 4. summarizes the cross-validation consistency and prediction error through multifactor dimensionality reduction for each mutation of high-difference SNP sites and host factors. “Rv0210-BMI” model had a maximum testing accuracy of 61.2% and a maximum cross-validation consistency (8/10, *P* < .0001). Figure 2 exhibits 3 combinations associated with high risk and low risk for the “Rv0210- Age groups-BMI” model. From the distribution of high and low-risk factors/SNP sites mutations, we can see that a strain with a mutation of Rv0210, and its host belonging to the normal BMI and low age group was more likely to be in cold spot area.

**Table 4:** The best model for predicting the likelihood of *M.* tb strains appearing in hot or cold spots.

Best Model	Training accuracy (%)	Testing accuracy (%)	CVC	*Χ* ^2^	*P* value
Rv0210	63.8	59.8	8/10	21.75	<.001
Rv0210, BMI	67.4	61.2	8/10	35.47	<.001
Rv0210, Age groups, BMI	70.1	60.5	4/10	44.04	<.001

CVC = cross-validation consistency

**Figure 3:**
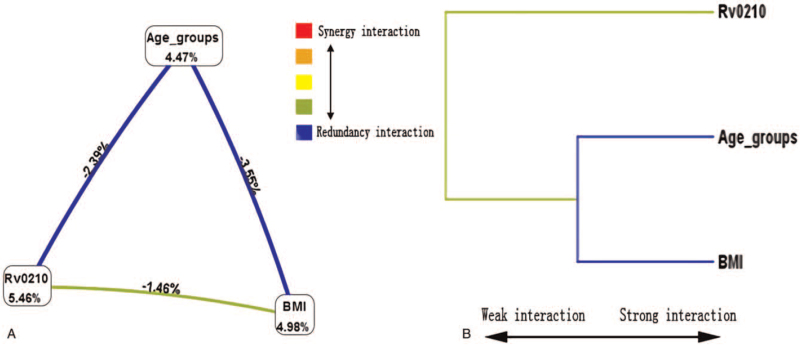
Hierarchical interaction graphs and interaction dendrograms. (A) For the hierarchical interaction graphs, the proportions at the bottom of each factor/mutation of SNP sites represent entropy, and the percentage on each line represents the interaction proportion of entropy between the 2 factors/mutations of SNP sites. The blue line represents redundancy interaction and the green line represents weak redundancy interaction. (B) For the interaction dendrograms, the red line represents synergy redundancy interaction and the blue line represents redundancy interaction. From left to right the interaction was more intensive.

A hierarchical interaction graph based on all alternative models is shown in Figure 3A. It displays a clear negative interaction effect of age groups with BMI (interaction entropy: −3.55%) and mutation of Rv0210 (interaction entropy: −2.39%). Through the mutations of Rv0210 and BMI had a low independent effect (interaction entropy: −1.46%).

An interaction dendrogram is shown in Figure 3B and shows that age groups and BMI were located on the same branch. These 2 factors were estimated to have the strongest redundancy interaction, as indicated visually by the blue line. The mutation of Rv0210 was on a different branch, demonstrating a weak redundancy interaction with other factors.

## 4 Discussion

During the period of this study, we collected most strains from areas with high and low reported incidence of tuberculosis. The data could therefore be representative of genetic and demographic diversity, at least to some extent.

In this study, Beijing family strains were the dominant genotype in both cold and hot spot areas as confirmed by whole genome sequencing. Beijing family strains, which were first reported in 1995, have spread worldwide.^[21]^ These strains originated in Beijing and Mongolia, have highly conserved spoligotyping patterns, and characteristic IS6110 RFLP patterns of Mtb isolates.^[22,23]^ In addition to the biological characteristics of high multidrug-resistance rate,^[24–26]^ the Beijing family strains may also have higher virulence compared to the other genotype strains. A study conducted in The Gambia suggested that patients infected with the Beijing family strain were more likely to progress to disease than those infected with *Mycobacterium africanum*.^[27]^ However, the sample size of that study was small and other *M. tb* complex genotypes among the control group were less clear. Interestingly, from animal models, there is clear evidence that the expression of proteins, glycolipids and triglycerides in the Beijing strain is altered, which may contribute to increased pathogenicity. In our study, the proportion of Beijing family strains in hot spots was significantly higher than that in cold spots (*P* < .05). This suggests that the virulence of strains in hot spots is higher than those in cold spots. In addition, previous investigations by our team have also found that the proportion of latent TB infection (The tuberculin test induration was greater than 10 mm in diameter) in close contacts of TB patients of hot spots areas was significantly higher than that in cold spots (35.7% vs 24.25, *P* < .05).^[20]^ It suggests that the strains from the hot spot areas may have a greater transmission.

In addition to the ratio of genetic makeup, there were also statistically significant differences in the distribution of age, BMI, history of TB patient contact and migration history between participants from cold and hot spots. A study performed in The Netherlands found that disease transmission was higher in younger aged people,^[28]^ a finding that contrasted with our study. In our study, the proportion of elderly TB cases was higher in areas with a high TB incidence. This phenomenon may be related to the socio-economic status of different regions. As an independent predictor of TB incidence, it remains to be seen whether socioeconomic factors or immune factors influence the spread and development of TB. We hypothesize that the high polymorphism of strains in cold spots might be related to the high frequency of traveling among this population.

Population genetic structure comparison is the main method used to determine whether 2 populations evolve independently or have gene exchange.^[29,30]^ Because *M. tb* is a highly conserved and differentiated species, the molecular structure differences of its mutant subgroups are relatively small. Through a comparison of population genetic structure differences, this study found that the average population structure difference coefficient of strains collected in hot spots and cold spots was only 0.019. In particular, the dominant strain (Beijing family) in this study is more evolutionary conserved than other *M. tb* lineages, and thus less likely to undergo recent mutations.^[31]^ However, some specific SNP locations showed significant population differences. The gene locus of these positions included Rv1186c, Rv3900c, Rv1508c, and Rv0210, where Rv1508c belongs to the fragment of DR4. It has been mentioned that the knock-in of RD4 can improve the protective efficacy of the BCG vaccine. However, the effect of this region deletion on the pathogenicity of *M. tb* and its clinical phenotype still needs further research. However, this study found that the mutation rate of Rv1508c in hot spots was higher than that in cold spots (58.5% vs 34.0%) which conflicts with other studies. However, after adjusting for other factors, the difference was not statistically significant. The proportion of Rv1186c mutation in hot spots was significantly lower than that in cold spots. The product of this gene is a conserved protein called PruC. *M.tb* is an obligate aerobic bacterium. However, it has shown remarkable metabolic flexibility, being able to survive and metabolize for a long time without oxygen.^[32]^ A study showed that M.tb can grow on carbon - and energy-derived proline under hypoxia conditions and is regulated by a unique transcription factor (PruC).^[33]^ Thus, mutations in this gene indicate the immune escape and changes in pathogenicity that affect the transmissibility of *M. tb*.

The global persistence of *M. tb* infection over a long time suggests that there is strong evolutionary pressure for the interaction between host and pathogen genomes.^[34–36]^ susceptibility gene polymorphisms interacted by M. tb play a role in the development of TB.^[37,38]^ The GG genotype of IL-17 rs2275913 in the Spanish population is associated with a high risk of tuberculosis,^[39]^ while the CC genotype of rs763780 in the Chinese population increases the risk of tuberculosis.^[40]^ In this study, the proportion of Zhuang population was significantly higher in TB hot spots. However, because the 2 research sites are not comparable, ethnicity was not included in the MDR model for interactive analysis. Nevertheless, further biochemical and immunological evidence is needed to confirm the hypothesis of high susceptibility among Zhuang population. Besides, the results of this study suggest that there is a strong negative interaction between age group and mutation of Rv0210. A similar result showed that SNP Rs9272785 for HLA-DQA1 showed a suggestive association in the young-onset Tuberculosis subgroup (onset age 20–40 years, N = 396), although no significant association was found in the entire sample.^[41]^ This result supports the hypothesis that the pathogenesis of TB strains in different age groups and that genetics may play an important role only in the younger onset of TB. In areas with high rates of TB, older patients showed significantly lower BMI and had a strong negative interaction. These results suggest the importance of immune level and nutrition to TB.^[42]^ Therefore, in addition to socioeconomic factors, we also need to consider the impact of nutritional deficiencies on TB development.

In conclusion, we found significant evidence for an association between the SNP difference of *M. tb*, host environment and TB epidemic. Our data suggest a statistically significant role of age, BMI and the polymorphisms of Rv0210 genes in the transmission and development of *M. tb*. The results provide clues for the study of susceptibility genes of *M. tb* in different populations. Strengthening genotype monitoring of strains in various regions can be used as an early warning signal of epidemic spillover (Supplemental Digital).

## Acknowledgments

We would like to thank all the health workers for their assistance in performing the survey. Statistical support and English grammar were revised from Edward McNeil, Prince of Songkla University, Songkhla, Thailand.

## Author contributions

**Conceptualization:** Zhezhe Cui, Jun Liu, Yue Chang, Dingwen Lin.

**Data curation:** Zhezhe Cui, Dan Luo.

**Formal analysis:** Zhezhe Cui, Jun Liu, Yue Chang, Dingwen Lin.

**Funding acquisition:** Zhezhe Cui, Dingwen Lin, Dan Luo.

**Investigation:** Zhezhe Cui, Dingwen Lin.

**Methodology:** Zhezhe Cui, Jun Liu.

**Resources:** Jing Ou, Liwen Huang.

**Software:** Zhezhe Cui, Jun Liu.

**Writing – original draft:** Zhezhe Cui, Jun Liu, Dingwen Lin.

## Supplementary Material

Supplemental Digital Content
