# Supplementary material for: Interaction analysis of Mycobacterium tuberculosis between the host environment and highly mutated genes from population genetic structure comparison
Source: Medicine (Baltimore). 2021 Sep 3;100(35):e27125. doi: 10.1097/MD.0000000000027125 (PMC8415957; doi:10.1097/MD.0000000000027125)
Supplement: Supplemental Digital Content [file medi-100-e27125-s001.pdf]

Structured questionnaire for index TB patients

Questionnaire for index TB patient
In_____county, Guangxi, China

Date: |__||__|/|__||__|/|__||__|
Starting time: |__||__|/|__||__|

Section 1 Background characteristics

1.1	Household code	___________(2 digits)	hc|__||__|
1.2	Resident	___________township	res |__|
1.3	Duration of living at resident	(1) ≥6 months (2)< 6 months	stay|__|
1.4	Sex	(1) Male (2) Female	gen |__|
1.5	Age (completed year)	___________year	age|__||__|
1.6	Ethnicity	(1) Han (2) Zhuang<br>(3) Miao (4) Yao<br>(5) Others (specify)___	eth|__|
1.7	Education level (passed level)	(1) None (2) Read and write<br>(3) Primary (4) Middle<br>(5) High (6) Graduate	edu|__|
1.8	Occupation	（0）No (1) Agriculture (2) Labor<br>(3) Government staff (4) Business/Employee<br>(5) Student (6) Unstable job<br>(7) Dependent (8) Others (specify)___	occy|__|
1.9	Marital status	(1) Single (2) Marriage<br>(3) Divorce (4) Separate<br>(5) Widowhood (6) Others (specify)___	ms|__|

1.10	Monthly payroll income (basic salary + bonus)	|__| 1. 3000 RMB and below<br>|__| 2. 3001-4000 RMB<br>|__| 3. 4001-5000RMB<br>|__| 4. 5001-6000RMB<br>|__| 5. 6001-7000 RMB<br>|__| 6. 7001-8000RMB<br>|__| 7. 8000 RMB above	inc |__|
1.11	Is there any history of TB in your family?	(0) No (1) Yes (Relation________)	ptbfam|__|
1.12	How many household members living with you?	___________members	m|__||__|

Section 2 Health related behaviors

2.1	Smoking	(1) Never smoked (Go to no.2.5)<br>(2) Smoked in the past (Go to no.2.4)<br>(3) Current smoker	psm|__|
2.2	If smoker, number of cigarettes per day	___________ cigarettes	pns|__||__|
2.3	Duration of smoking	___________ years	pds|__||__|
2.4	If ex-smoker, when stopped smoking?	___________ years	pssm|__||__|
2.5	Passive smoker	(1) No (2) Few times in week<br>(3) Very few times in week<br>(4) Very few times daily<br>(5) Few times daily<br>(6) Most of the times daily	pps|__|

2.6	Drinking	(1) Never drink (Go to no. 2.11)<br>(2) Drink in the past (Go to no. 2.10)<br>(3) Current drinker	pdrink|__|
2.7	Duration of drinking	___________years	pddr|__||__|
2.8	If current drinker, frequency of drinking	(1) Daily (2) Almost daily<br>(3) 3-4 days/week (4) 1-2 days/week<br>(5) 2-3 times/month (6) Once a month	pfdrink|__|
2.9	Capacity of alcohol	______ml/one time	capal|__||__||__|
2.10	If ex-drinker, when stopped drinking?	___________years	psdr|__||__|
2.11	HIV status	(1) Positive (2) Negative (3) Unknown	hiv |__|
2.12	Other immunocompromising diseases	___________	oid |__|
2.13	DM status	(1) Yes (2) No (3) Unknown	dm |__|
2.14	BCG status	(1) Yes (2) No (3) Unknown	bcg |__|
2.15	Hight	_______cm	bh|__|
2.16	Weight	_______kg	bw|__|
2.17	House style	(1) Flat (2) cement dwelling (3) Dirt room	hs|__|
2.18	House area	_______m^2	har|__|
2.19	Household contact in 6 months	(1) Yes (2) No (3) Unknown	cc |__|
2.20	Daily activity scale	___________	das |__|
2.21	Travel history in 2 years	___________	thty|__|

Section 3 History taking about TB before diagnosis

3.1	Any signs and symptoms of TB before TB diagnosis? (multiple answers allowed, with probe)	(1) Cough >2 weeks<br>(2) Blood in sputum<br>(3) Fever >2 weeks<br>(4) Weight loss<br>(5) Chest pain<br>(6) Others (specify)______	pcc|__| pbld|__| pfev|__| pwt|__| pcpain|__| poths|__|
3.2	Frequency of coughing before TB treatment	(1) No cough (Go to no.3.6)<br>(2) Mild, seldom cough<br>(3) Moderate, often cough but not disturb for resting<br>(4) Sever, cough disturb for resting	pfcou |__|
3.3	How many day have you got coughing before TB treatment?	____________days	pdcou|__|
3.4	Have you ever been got any TB treatment?	(0) No (Go to no.3.6) (1) Yes	pretreat|__|
3.5	If yes, how many times treated for TB?	____________number	pnretr|__|
Record review			
3.6	AFB for sputum smear	(1) +1 (2) +2 (3) +3 (4) +4	pafb|__|
3.7	Site of TB	(1) Pulmonary (2) Extra-pulmonary TB	ptbsite|__|
3.8	Retro status	(1) Positive (2) Negative (3) Unknown	pretro|__|
3.9	Treatment regime	(1) Cat I (2) Cat II (3) Cat III	ptreat|__|
3.10	Type of Health Insurance	(1) the new rural cooperative medical system<br>(2) medical insurance for urban workers	thi|__|

		3) Urban residents' basic medical insurance<br>(1) Other	
3.11	Duration of diagnosis delay	___________months	dodd|__|

Section 4 Time taken and distance from healthcare provider residence to TB patient

4.1	Time taken	___________minutes	ptrt|__||__|
4.2	Distance	___________kilometers	ptrdi|__||_|

Thank you for your participation

End time: |__||__|/ |__||__|

Signature of data collector_________
